# Persistence and predictors of self-injurious behaviour in autism: a ten-year prospective cohort study

**DOI:** 10.1186/s13229-019-0307-z

**Published:** 2020-01-20

**Authors:** Catherine Laverty, Chris Oliver, Jo Moss, Lisa Nelson, Caroline Richards

**Affiliations:** 1 School of Psychology, University of Birmingham, Birmingham, B15 2TT UK; 2 Cerebra Centre for Neurodevelopmental Disorders, School of Psychology, University of Birmingham, Birmingham, B15 2TT UK; 3 School of Psychology, University of Surrey, Guildford, Surrey GU2 7XH UK

**Keywords:** Autism, Impulsivity, Prevalence, Risk marker, Self-injury, Self-restraint

## Abstract

**Background:**

Self-injurious behaviours, such as head banging, hair pulling, skin picking and scratching, are common in individuals with autism. Despite high prevalence rates, there is a paucity of longitudinal research to refine models of risk and mechanism and inform service planning. In this longitudinal study, we investigated self-injury in a cohort of individuals with autism over 10 years to identify behavioural and demographic characteristics associated with persistent self-injury.

**Methods:**

Carers of 67 individuals with autism completed questionnaires relating to the presence of self-injury and relevant risk markers at *T*_1_ (mean [SD] age in years 13.4 [7.7]) and *T*_3_ (mean [SD] age in years 23.9 [7.7]) 10 years later. Forty-six of these also took part at *T*_2_ (3 years after initial participation). Analysis assessed demographic and behavioural risk markers for self-injury, as well as the predictive value of items assessed at *T*_1_and *T*_2._

**Results:**

Self-injury was persistent in 44% of individuals over the 10-year period, with behavioural characteristics of impulsivity (*p* < .001) and overactivity (*p* = .002), identified as risk markers for persistence. A predictive model of self-injury was derived from LASSO analysis, with baseline impulsivity, interest and pleasure, stereotyped behaviour, social communication and adaptive functioning predicting self-injury over 10 years.

**Conclusions:**

In this unique longitudinal investigation into the persistence of self-injury in a non-clinical sample of individuals with autism over a 10 year period, we have identified a novel, robust and stable profile of behavioural characteristics associated with persistent self-injury. Findings support an early intervention strategy targeted towards individuals identified to be at a higher risk of developing self-injurious behaviour.

## Background

Self-injurious behaviour (SIB), defined as a physical non-accidental act of producing injury to one’s body [1], encompasses behaviours such as head banging, hair pulling and skin picking [2]. In addition to the direct negative physical consequences of SIB, the presence of SIB increases the risk of family, educational and residential placement breakdowns [3], restrictive practices in primary care settings [4] and use of psychotropic medications [5]. Whilst SIB is detrimental to the individual and those around them, limited epidemiological data exist delineating the developmental trajectory of these behaviours. Given the significant financial burden for service providers [6] and noted lack of interaction with professionals to alleviate behaviours at the individual level [7], early intervention arguments to ameliorate the development of SIB are growing. It is imperative that mechanisms underpinning SIB are understood to optimise the value of such strategies.

Prevalence of SIB in individuals with autism is reported to be as high as 50% [8]; significantly higher than that for individuals with intellectual disability (12%) [9, 10]. Strikingly, the presence of characteristics associated with autism are associated with a higher prevalence of SIB [11] in multiple genetic syndromes indicating that both the presence of diagnosable autism *and* the presence of autism characteristics (such as stereotyped behaviour, insistence on sameness and repetitive use of language) elevate risk even in the highest risk groups [8, 11]. Prospective longitudinal cohort studies are required to explore the characteristics underpinning SIB, to ultimately reduce poor population outcomes for those with autism characteristics.

Current research demonstrates persistence of SIB across the life span [12], with one review suggesting SIB is both common and stable over time for individuals with autism [13]. Further studies also highlight persistence in adolescents and adults without autism and with broader developmental disabilities [14, 15]; however, further research is needed to extend this evidence. With research focussing on the development of SIB in clinical samples over short periods of time [9, 10, 16, 17], the naturalistic and longitudinal trajectory of SIB over extended periods of time remains unexplored. Cross-sectional data in people with intellectual disability contradicts the assumption of linear persistence, demonstrating a peak in SIB towards late adolescence before a fragmented decline with age [18]. Longitudinal research in autism is necessary to delineate purported age-related changes and describe the naturalistic developmental trajectory of SIB in a non-clinical sample.

Research provides evidence for demographic and behavioural risk markers associated with the presence of SIB [8, 17, 19, 20] that inform theoretical models. Historically, operant models explaining the maintenance of SIB have prevailed [21], yet such models do not consider the importance of individual characteristics, instead suggesting equal risk across individuals. Oliver and Richards proposed an extended model, integrating childhood characteristics which account for variability in both the presence of SIB and developmental trajectory [22]. Identification of demographic and behavioural markers as robust variables associated with the presence, severity and persistence of SIB in autism would lend further support to this model and implicate potential causal mechanisms driving poor clinical outcomes.

Overactivity and impulsivity have consistently been identified as behavioural characteristics associated with the presence of SIB [22]. Within multiple samples of individuals with autism, overactivity and impulsivity predict both the presence and severity of SIB [23–25], with emerging evidence suggesting that these characteristics also predict persistence [24]. Research further highlights this association amongst samples with genetic syndromes [21]. Importantly, overactivity and impulsivity are known behavioural markers for impairments in behavioural inhibition. Behavioural inhibition comprises both the capacity to inhibit prepotent responses to evoking stimuli and the capacity to inhibit a response once emitted [26–28]. Thus, the association between impulsivity/overactivity and self-injury alludes to a fundamental cognitive vulnerability which may act as a mechanism interacting with operant learning to drive both the presence and severity of SIB [22].

Concomitant evidence for this hypothesis is the presence of self-restraint. Self-restraint behaviours are those which restrict the movement of an individual’s body parts using clothing, objects or a person’s own body [29, 30]. Self-restraint is significantly more common in individuals with self-injury [31] and is described as serving the purpose of inhibiting severe SIB [29, 31]. The presence of these behaviours suggests that environmental and sensory contingencies alone are insufficient to constrain self-injury. Description of the putative association between SIB and self-restraint in a prospective longitudinal at-risk cohort, such as those with autism, will provide a useful context in which to evaluate the hypothesised model implicating impaired behavioural inhibition as a risk marker.

In summary, SIB leads to significant physical, financial and emotional cost for individuals and caregivers. A paucity of research has evaluated persistence of SIB in autism. Whilst current data support the cross-sectional associations of behavioural characteristics such as overactivity and impulsivity with SIB, there is little evaluation of these associations longitudinally. A prospective longitudinal cohort affords the opportunity to describe and evaluate the presence, persistence and predictive associations with SIB in autism. Time 1 data (*T*_1_) and subsequent 3-year follow up (*T*_2_) of this prospective cohort identified behavioural risk markers for persistent SIB within the current sample of individuals with autism [8, 24]. The present study (*T*_3_) extends the longitudinal study, investigating the persistence of SIB over 10 years. The following hypotheses are made:

1. SIB will be persistent between *T*_1_ and *T*_3_ for the majority of individuals
2. Higher levels overactivity and impulsivity at *T*_3_ will be positively associated with the following:
  - The presence of self-injurious behaviour at *T*_3_
  - The presence of self-restraint behaviours at *T*_3_
3. Higher levels overactivity and impulsivity at *T*_2_ will predict longitudinally the presence of self-injurious behaviour at *T*_3_
4. Higher levels overactivity and impulsivity at *T*_1_ will predict longitudinally the following:
  - The presence of self-injurious behaviour at *T*_3_
  - The presence of self-restraint behaviours at *T*_3_

## Method

### Participants

At Time 1 (*T*_1_) participants were recruited through the National Autistic Society [8]. All participants who consented to future contact were invited to take part in the present study (*N* = 241), independent of participation at the Time 2 (*T*_2_) follow- up [24]. Seventy-two participants completed the study (return rate: *T*_2_ = 35.78%, *T*_3_ = 29.58%). Participants were excluded if (a) they were under the age of four at *T*_1_, (b) they did not have a diagnosis of autism confirmed by a relevant professional^1^, (c) they had a diagnosis of a genetic syndrome and (d) they scored above the autism threshold on the Social Communication Questionnaire on fewer than two of the three time points. Five participants were therefore excluded, leaving a final sample of 67.

### Procedure

Information packs containing an invitation letter and link to the online survey were sent to all participants. Using unique identifiers, participants completed the relevant consent forms, before being directed through each measure and submitting responses. Paper versions of questionnaires were available upon request. All participants were sent individual feedback reports upon completion of data analysis, detailing responses from participation in *T*_1_, *T*_2_ and *T*_3_ studies. Ethical approval for this study was obtained from the ethical review committee at the University of Birmingham.

### Measures

The following questionnaires, suitable for carer report in individuals with intellectual disabilities, were included:

A demographic questionnaire detailing person characteristics, age, mobility and verbal ability was used. Inclusion allowed for the assessment of potential associations demographic characteristics may have in subsequent self-injury analysis. A service receipt sub-section was also included, detailing clinical services accessed over the 10-year period, and carer’s evaluation of their utility.

The Wessex was used to assess self-help adaptive functioning [32]. The questionnaire has been shown to be successful in measuring ability amongst those with an intellectual disability and has good inter-rater reliability at the subscale and item levels [33]. Inclusion allowed for exploration of how individual adaptive functioning had developed since *T*_1_.

The Activity Questionnaire (TAQ) assessed impulsivity and overactivity [34]. It consists of three subscales, and cut offs are established to account for unusually high scores [34]. The measure has been shown to have good inter-rater reliability (mean .56), test-retest reliability (mean .75) with assessments of internal consistency showing all subscales correlate to a moderate degree [35] Impulsivity was associated with persistent self-injury at *T*_2_ analysis [24], with current analysis therefore exploring the development of this association.

The Social Communication Questionnaire (SCQ) was used to assess behaviours associated with autism within the sample [36]. The measure demonstrates good concurrent validity (ADOS [37]; ADI-R [38]), and internal consistency (*α* = .90 for the total scale). It is a non-diagnostic screening tool and was used to exclude participants at *T*_1._ The measure has a recommended cut-off score of 15 [36], although it is argued this benchmark should not be rigid and can vary based upon individual characteristics and severity of symptoms [39]. Thus, as all participants had a clinical diagnosis of autism, participants were only excluded from *T*_3_ analysis if they scored below this cut off on more than two data collection points. Given the longitudinal nature of the study, the lifetime SCQ was used to collect data at *T*_2_ and *T*_3_, measuring individual change over time.

The Repetitive Behaviour Questionnaire (RBQ) was used to rate frequency of repetitive behaviour and severity on a Likert scale [40]. Repetitive behaviours are considered to be a risk marker for self-injury [41, 42]. It was therefore considered a relevant measure to include, exploring how such behaviours develop with age. The measure has been shown to have good reliability in a sample of individuals with heterogeneous causes of intellectual disability [35]. Concurrent, content and face validity has also been evidenced and shown to be robust [35].

The Challenging Behaviour Questionnaire (CBQ) evaluated self-injury, aggression, destruction of property and stereotyped behaviour within the past month [43]. The questionnaire allows for topographies and severity of SIB to be described. Analysis of psychometric properties has found good inter-rater reliability [43].

In addition to measures assessed at *T*_1_ [8], The Self-Restraint Questionnaire was included at *T*_3_ [30]. Self-restraint behaviours are described to serve the purpose of inhibiting severe SIB [23]. The measure describes seven topographies of self-restraint, with a checklist to indicate any behaviour present. The measure has been shown to be reliable with fair inter-rater agreement across all items, and good reliability on three of the subscales [35]. Validity has also been evidenced through a series of direct observations (89.6% across observation and scores) [35].

### Data analysis

Normality of data was assessed using Kolmogorov-Smirnov tests. Due to the dataset significantly deviating from normal distributions (*p* < .05), non-parametric analyses were employed. Mann-Whitney *U* tests were conducted to assess demographic differences between those who took part in *T*_3_ study and those who declined to take part, to evaluate how representative the *T*_3_ sample was of the original *T*_1_ sample. Chi-Square and relative risk statistics were conducted to assess service use between those presenting with self-injury at *T*_3_, and those who did not. Chi-Square and Mann-Whitney *U* analyses were also used to explore demographic and behavioural differences between those who showed SIB at *T*_3_ and those who did not. McNemar analyses were conducted to assess persistence and topographies of self-injury from *T*_2_ to *T*_3_ and *T*_1_ to *T*_3_. Kruskal-Wallis analyses were used to evaluate putative risk markers between *T*_2_ and *T*_3_, whereby participants were split into absent (self-injury absent at both *T*_2_ and *T*_3_), transient (self-injury absent at either *T*_2_ or *T*_3_) and persistent (self-injury present at both *T*_2_ and *T*_3_) groups. This analysis was also repeated for data collected at *T*_1_ to *T*_3_, data was again split into three groups: absent (self-injury absent at both *T*_1_ and *T*_3_), transient (self-injury absent at either *T*_1_ or *T*_3_) and persistent (self-injury present at both *T*_1_ and *T*_3_) groups. Pairwise post hoc Mann-Whitney *U* analyses corrected for multiple comparisons were used to assess significant differences between the categorical groups. Kruskal-Wallis analyses were also used to explore putative risk markers associated with self-restraint at *T*_3_. In order to summarise data collected at each of the three time points and clearly depict behavioural characteristics that cross-sectionally and longitudinally predicted SIB, standardised effect sizes were calculated. Data from *T*_1_ [8] and *T*_3_ [24] were reassessed, and *Z* scores were extracted, with standardised effect sizes then calculated. Finally, to explore the predictive value of items assessed at *T*_1_, least absolute shrinkage and selection operator (LASSO) analysis was conducted, with the outcome variables being the presence of self-injurious or self-restraint behaviour at *T*_3_. LASSO analysis was chosen as evaluation of variance inflation factors indicated high levels of multicollinearity within the predictor variables, violating assumptions of traditional regression analysis [44]. As LASSO analysis is a penalised form of regression, poorer parameters are reduced where there is multicollinearity, minimising over-prediction in smaller samples [45]. Analysis utilised R software for statistical computing (version 3.5), operating the ‘glmnet’ package [46].

## Results

### Demographic characteristics of the sample

In order to ensure those who participated at *T*_3_ were representative of the original *T*_1_ sample, comparisons were made between those who took part at *T*_3_ and those who declined on a range of demographic and behavioural characteristics from *T*_1_. The data in Table 1 reveal that those who took part at *T*_3_ did not significantly differ from those who declined to take part on any of the demographic measures collected at *T*_1_. However, differences were obtained for some measures of behavioural characteristics. Individuals who took part at *T*_3_ showed significantly lower levels of activity, impulsivity, compulsive behaviour and restricted preferences. They also displayed higher levels of repetitive behaviour. The final sample was not significantly different regarding levels of self-injury and was therefore deemed representative of the *T*_1_ sample for the purposes of the present study. Demographic characteristics of parents and caregivers that participated at *T*_3_ are also presented (Table 2) detailing self-reported levels of education and household income.

**Table 1:** Demographic and behavioural characteristics of those who participated at *T*_3_ and those who declined to take part *T*_3_

		Took part *T*_3_ *N* = 67	Declined to take part *T*_3_ *N* = 205	Mann-Whitney *U*/*χ*^2^	*p* value
Age	Median (IQR)	12 (8)	10 (7.75)	5682	.055
Gender	% male	80.6% (54)	87% (176)	.405	.525
Ability	% partially able/able	94% (63)	90% (183)	6110	.173
Mobility	% mobile	97% (65)	97% (197)	N/A^a^	1.000
Speech	% verbal	91% (61)	86% (174)	1.097	.295
Self-injury	% with behaviour	37.3% (25)	37% (76)	.004	.949
Mood total score	Median (IQR)	35 (9)	33 (10)	6472	.593
Activity total score	Median (IQR)	32 (31)	39 (32)	5475	.037*
TAQ impulsivity	Median (IQR)	15 (12)	17 (10)	5613	.046*
TAQ overactivity	Median (IQR)	14 (14)	16 (18)	5758	.076
Repetitive behaviour total score	Median (IQR)	35 (9)	28 (25)	4734	< .001*
RBQ compulsive behaviour	Median (IQR)	5 (7)	6 (11)	5462	.046*
RBQ insistence on sameness	Median (IQR)	3 (6)	4 (5)	5667	.181
RBQ restricted preferences	Median (IQR)	3 (5)	5 (5)	3988	.004*
RBQ repetitive use of language	Median (IQR)	6 (6)	6 (7)	4669	.200
Autism phenomenology total score	Median (IQR)	25 (10)	25 (11)	5462	.628

^a^Fisher’s exact was calculated where 50% expected count < 5, *p < .05

**Table 2:** Educational and financial characteristics of parents and caregivers of those who participated at *T*_3_ (67)

Fewer than 5 GCSE’s or O Level’s (grades A-C), NVQ 1 or BTEC First Diploma	7.5 (5)
5 or more GCSE’s or O Level’s (grades A-C), NVQ 2 or equivalent	20.9 (14)
3 or more ‘A’ Levels, NVQ 3, BTEC National or equivalent	14.9 (10)
Polytechnic/University degree, NVQ 4 or equivalent	40.3 (27)
Masters/Doctoral degree, NVQ 5 or equivalent	14.9 (10)
Less than £15,000^2^	10.8 (7)
£15,001 to £25,000	20 (13)
£25,001 to £35,000	10.8 (7)
£35,001 to £45,000	12.3 (8)
£45,001 to £55,000	9.2 (6)
£55,001 to £65,000	7.7 (5)
£65,001 or more	29.2 (19)

^1^All results displayed as % (*N*)

^2^For data regarding financial income, total sample = 65 due to two cases of missing data

### Persistence of self-injury

In order to assess hypothesis 1 and 2, the persistence and stability of SIB was explored. Groups were first created based upon the presence of self-injury at *T*_2_ and *T*_3_: Absent, Remission, Incidence and Persistent. McNemar analysis was employed to explore significant differences between groups. Percentages of participants showing self-injury and individual topographies of self-injury were calculated for each of these groups (Table 3). Analysis showed no significant change in self-injury between these time points.

**Table 3:** Percentage (*N*) of participants showing remission, incidence, persistent or absent self-injurious behaviour between *T*^2^ and *T*^3^

Behaviour	Absent (Absent 2010, absent 2017)	Remission (Present 2010, absent 2017)	Incidence (Absent 2010, present 2017)	Persistent (Present 2010, present 2017)	*P* (2-tailed)	Remission in participants with self-injury at *T*_1_	Persistence in participants with self-injury at *T*_1_
Self-injury	63 *(29)*	9 *(4)*	4 *(2)*	24 *(11)*	.687	27 (4)	73 (11)
Hits self with body	80 *(37)*	4 *(2)*	7 *(3)*	9 *(4)*	1.000	33 (2)	67 (4)
Hits self against object	83 *(38)*	4 *(2)*	7 *(3)*	7 *(3)*	1.000	40 (2)	60 (3)
Hits self with object	98 *(45)*	0 *(0)*	0 *(0)*	2 *(1)*	1.000	-	-
Bites self	78 *(36)*	13 *(6)*	2 *(1)*	7 *(3)*	.125	67 (6)	33 (3)
Pulls self	91 *(42)*	4 *(2)*	0 *(0)*	4 *(2)*	.500	50 (2)	50 (2)
Rubs/scratches self	91 *(42)*	4 *(2)*	0 *(0)*	4 *(2)*	.500	50 (2)	50 (2)
Inserts	-	-	-	-	-	-	-

This analysis was repeated for data collected at *T*_1_–*T*_3_ (Table 4). The data in Table 3 show significant reductions in the presence of self-injury (*p* = .031), and the specific topography of self-biting (*p* = .039) from *T*_1_ to *T*_3_. Self-injury remitted in 56% of individuals displaying SIB at *T*_1_ but was persistent in 44% of individuals over 10 years. There were no other significant differences within individual topographies of self-injury.

**Table 4:** Percentage (N^a^) of participants showing remission, incidence, persistent or absent self-injurious behaviour between *T*^1^ and *T*^3^

Behaviour	Absent (Absent 2007, absent 2017)	Remission (Present 2007, absent 2017)	Incidence (Absent 2007, present 2017)	Persistent (Present 2007, present 2017)	*P* (2-tailed)	Remission in participants with self-injury at *T*_1_	Persistence in participants with self-injury at *T*_1_
Self-injury	56 *(37)*	21 *(14)*	6 *(4)*	17 *(11)*	.031*	56 (14)	44 (11)
Hits self with body	76 *(50)*	11 *(7)*	6 *(4)*	8 *(5)*	.549	58 (7)	42 (5)
Hits self against object	89 *(59)*	2 *(1)*	5 *(3)*	5 *(3)*	.625	25 (1)	75 (3)
Hits self with object	-	-	-	-	-	-	-
Bites self	82 *(54)*	12 *(8)*	2 *(1)*	5 *(3)*	.039*	72.7 (8)	27.3 (3)
Pulls self	91 *(60)*	5 *(3)*	3 *(2)*	2 *(1)*	1.000	75 (3)	25 (1)
Rubs/scratches self	83 *(55)*	11 *(7)*	6 *(4)*	0 *(0)*	.549	100 (7)	0 (0)
Inserts	-	-	-	-	-	-	-

^a^Missing data from *T*_1_ reduces analysis sample to *N* = 66

*p < .05

In order to explore any mediating effect of service use upon persistence of SIB at *T*_3_, Chi-squared analysis with accompanying relative risks were calculated (see Table 5). Results show that there were significant differences between the four groups (persistent, absent, remitted and incident SIB) regarding access to paediatricians (*χ*^2^ (2) = 12.765, *p* = .002). Post hoc analysis showed both the persistent and transient group accessed paediatricians more than the absent group (*p* < .001). There were no other significant differences regarding service providers, and relative risk analysis comparing absent and persistent group revealed no significant differences.

**Table 5:** Number and percentage of individuals with autism spectrum disorder accessing services and Chi-squared analysis

	Absent (No SIB *T*_1_ or *T*_3_) *N* = 37	Transient (SIB at either *T*_1_ or *T*_3_) *N* = 18	Persistent (SIB *T*_1_ and *T*_3_) *N* = 11	Chi-squared test		
				*χ*^2^	df	*p* value
GP	34 (92%)	16 (89%)	10 (91%)	0.132	2	.936
Psychiatrist	12 (32%)	9 (50%)	4 (36%)	1.601	2	.449
Clinical psychologist	11 (30%)	9 (50%)	1 (9%)	5.436	2	.066
Occupational therapist	9 (24%)	5 (28%)	6 (55%)	3.741	2	.154
Speech and language therapist	11 (30%)	9 (50%)	6 (55%)	3.353	2	.187
Support group	10 (27%)	6 (33%)	4 (36%)	0.458	2	.795
Social worker	19 (51%)	10 (57%)	9 (82%)	3.264	2	.196
Nurse	8 (22)	5 (28%)	5 (45%)	2.431	2	.297
Paediatrician^a^	2 (5%)	7 (40%)	5 (45%)	12.765	2	.002*

^a^Post hoc analysis showed both the persistent and transient group accessed paediatricians more than the absent group (*p* < .001)

*p < .05

In summary, the analyses support the null hypothesis as results show a significant reduction in self-injury within the sample over the longitudinal period.

### Demographic and behavioural variables associated with the presence of self-injury and self-restraint

In order to assess hypothesis 4*,* analysis explored *T*_3_ demographic and behavioural variables associated with the presence of self-injury and self-restraint at *T*_3_. This analysis allows for insight into the presence of risk marker associated with behaviours cross-sectionally. Participants were grouped based upon the presence or absence of self-injury or self-restraint behaviours at *T*_3_. Chi-square, Fisher’s exact test’s and Mann-Whitney *U* analyses were conducted to compare scores between those with present versus absent self-injury at *T*_3_ (Table 6) and self-restraint at *T*_3_ (Table 7).

**Table 6:** *T*_3_ demographic and behavioural characteristics for participants with and without self-injury at *T*_3_

		Individuals with self-injury *T*_3_ (*N* = 16)	Individuals without self-injury *T*_3_ (*N* = 51)	Chi-square/Mann-Whitney *U*	*p* value	Effect size
Gender	Male; percentage (*N*)	81 (13)	80 (41)	N/A^a^	1.00	
Ability	Partially able/able; percentage (*N*)	100 (16)	96 (49)	N/A^a^	1.00	
Mobility	Mobile; percentage (*N*)	100 (16)	96 (49)	N/A^a^	1.00	
Speech	Verbal; percentage (*N*)	81 (13)	94 (48)	N/A^a^	.142	
Mood total score	Median (IQR)	38 (9)	36 (11)	357	.448	
Mood	Median (IQR)	20 (3)	20 (5)	394	.830	
Interest and pleasure	Median (IQR)	17 (6)	15 (7)	353	.417	
Activity total score	Median (IQR)	43 (29)	18 (22)	187	< .001*	0.4
Impulsivity	Median (IQR)	19 (11)	10 (9)	187	< .001*	0.4
Overactivity	Median (IQR)	21 (20)	5 (9)	176	< .001*	0.4
Repetitive behaviour total score	Median (IQR)	26 (28)	14 (17)	228	.008*	0.3
Compulsive behaviour	Median (IQR)	9 (13)	5 (7)	275	.051	
Insistence on sameness	Median (IQR)	5 (4)	3 (5)	224	.006*	0.3
Stereotyped behaviour	Median (IQR)	7 (11)	3 (8)	286	.068	
Autism phenomenology total score	Median (IQR)	23 (14)	17 (12)	244	.015*	0.3
Communication	Median (IQR)	8 (4)	7 (3)	345	.346	
Social interaction	Median (IQR)	9 (7)	5 (5)	281	.060	

^a^Fisher’s exact was calculated where 50% expected count < 5

*p < .05

**Table 7:** *T*_3_ Demographic and behavioural characteristics for participants with and without self-restraint at *T*_3_

		Individuals with self-restraint *T*_3_ (*N* = 29)	Individuals without self-restraint *T*_3_ (*N* = 38)	Chi-square/Mann-Whitney *U*	*p* value	Effect size
Gender	Male; percentage (*N*)	79 (23)	82 (31)	N/A^a^	1.000	
Ability	Partially able/able; percentage (*N*)	97 (28)	97 (37)	N/A^a^	1.000	
Mobility	Mobile; percentage (*N*)	100 (29)	95 (36)	N/A^a^	.502	
Speech	Verbal; percentage (*N*)	86 (25)	95 (36)	N/A^a^	.391	
Mood total score	Median (IQR)	33 (11)	38 (8)	364	.018*	0.3
Mood	Median (IQR)	19 (4)	21 (4)	348	.010*	0.3
Interest and pleasure	Median (IQR)	14 (7)	17 (6)	384	.034*	0.3
Activity total score	Median (IQR)	31 (29.5)	17 (25)	282	< .001*	0.4
Impulsivity	Median (IQR)	15 (12)	10 (14)	304	.002*	0.4
Overactivity	Median (IQR)	13 (17.5)	4 (11)	293	< .001*	0.4
Repetitive behaviour total score	Median (IQR)	21 (20)	15 (18)	389	.040*	0.3
Compulsive behaviour	Median (IQR)	6 (9.5)	6 (10)	478	.354	
Insistence on sameness	Median (IQR)	4 (4)	3 (5)	396	.047*	0.2
Stereotyped behaviour	Median (IQR)	7 (11)	3 (7)	402	.055	
Autism phenomenology total score	Median (IQR)	20 (10.5)	16 (11)	359	.015*	0.3
Communication	Median (IQR)	7 (4.5)	7 (4)	411	.074	
Social Interaction	Median (IQR)	7 (6.5)	5 (6)	433	.132	

^a^Fisher’s exact was calculated where 50% expected count < 5, *p < .05

Results in Table 6 show no significant differences between the presence of self-injury at *T*_3_ and demographic measures collected at *T*_3_. Total activity scores (*U* = 187, *Z* = − 3.259, *p* < .001, *r*^2^= 0.4) and subscales of overactivity (*U* = 176, *Z* = − 3.418, *p* < .001, *r* = 0.4) and impulsivity (*U* = 187, *Z* = − 3.264, *p* < .001, *r* = 0.4) were significantly higher for the self-injury group. Significant differences were also found on total repetitive behaviour scores (*U* = 228, *Z* = − 2.657, *p* = .008, *r* = 0.3), and insistence on sameness subscale (*U* = 224, *Z* = − 2.734, *p* = .006, *r* = 0.3), with the self-injury group scoring higher. Total autism characteristics group mean scores (*U* = 244.4, *Z* = − 2.422, *p* = .015, *r* = 0.3) were also significantly higher for the group showing self-injury at *T*_3_.

Results in Table 7 show no significant differences between those showing self-restraint at *T*_3_ and demographic measures collected at *T*_3_. Mood total score (*U* = 364, *Z* = − 2.371, *p* = .018, *r* = 0.3) and subscales of mood (*U* = 348, *Z* = − 2.591, *p* = .010, *r* = 0.3) and interest and pleasure (*U* = 384, *Z* = − 2.120, *p* = .034, *r* = 0.3) were significantly lower amongst those that showed self-restraint. Total activity scores (*U* = 282, *Z* = − 3.412, *p* < .001, *r* = 0.4) and subscales of overactivity (*U* = 293, *Z* = − 3.278, *p* < .001, *r* = 0.4) and impulsivity (*U* = 304, *Z* = − 3.139, *p* = .002, *r* = 0.4) were significantly higher within the self-restraint group. Repetitive behaviour total scores (*U* = 389, *Z* = − 2.052, *p* = .040, *r* = 0.3) and insistence on sameness (*U* = 396, *Z* = − 1.988, *p* = .047, *r* = 0.2), were also significantly higher amongst those who showed self-restraint. Finally, autism characteristics total score (*U* = 359, *Z* = − 2.439, *p* = .015, *r* = 0.3) was also significantly higher for individuals displaying self-restraint.

In summary, analyses support hypothesis 4, with behavioural measures of overactivity and impulsivity, alongside other behavioural characteristics, being significantly associated with both the presence of self-injury and self-restraint at *T*_3_.

### Longitudinal risk markers for the presence of self-injury and self-restraint behaviours

In order to assess hypothesis 3 and evaluate putative risk markers in those with self-injury compared to those without, participants were categorised into three groups: absent (self-injury absent at both *T*_2_ and *T*_3_; *N* = 11), transient (self-injury absent at either *T*_2_ or *T*_3_; *N* = 6), and persistent (self-injury present at both *T*_2_ and *T*_3_; *N* = 29). *T*_2_ behavioural characteristics were assessed across the three groups (for brevity, these data are presented in the appropriate column in Table 8). Kruskal-Wallis analyses identified significant differences between groups on measures of impulsivity (*χ*^2^ (2) = 9.705, *p* = .008) and overactivity (*χ*^2^ (2) = 9.764, *p* = .005). Differences were also found for insistence on sameness (*χ*^2^ (2) = 6.994, *p* = .030), restricted repetitive and stereotyped behaviours (*χ*^2^ (2) = 7.102, *p* = .0.29) and reciprocal social interaction (*χ*^2^ (2) = 7.185, *p* = .028). Pairwise post hoc analysis corrected for multiple comparisons revealed significant differences between scores in the absent and persistent self-injury groups for all behavioural variables.

**Table 8:** Effect sizes for cross-sectional and longitudinal behavioural risk markers of self-injury over ten years

	Cross-sectional effect sizes: significant differences between absent/present SIB			Longitudinal effect sizes: significant difference between absent/persistent SIB		
Risk markers	Time 1^1^ *N* = 149	Time 2^2^ *N* = 67	Time 3 *N* = 67	*T*_1_–*T*_2_ 3 years *N* = 67	*T*_2_–*T*_3_ 7 years *N* = 46	*T*_1_–*T*_3_ 10 years *N* = 67
Mood Interest and Pleasure Questionnaire						
Mood	O	O	O	O	O	O
Interest and pleasure	O	O	O	O	O	O
The Activity Questionnaire						
Impulsivity	**+**	**++**	**++**	**++**	**++**	**+++**
Overactivity	**+**	**++**	**++**	O	**++**	**++**
Impulsive speech	**+**	O	O	O	**++**	O
The Repetitive Behaviour Questionnaire						
Compulsive behaviour	O	**++**	O	O	O	O
Insistence on sameness	O	O	**++**	O	**++**	O
Stereotyped behaviour	O	**+**	O	O	O	O
The Social Communication Questionnaire						
Communication	O	O	O	O	O	O
Social interaction	O	**++**	O	**++**	**++**	O
Repetitive behaviour	O	**++**	**++**	O	**++**	O

Effect sizes are *R* interpreted with Cohens *D* (‘O’, none, ‘**+**’, small, ‘**++**’, medium, ‘**+++**’, large)

1[8]

2[23]

**Fig. 1:**
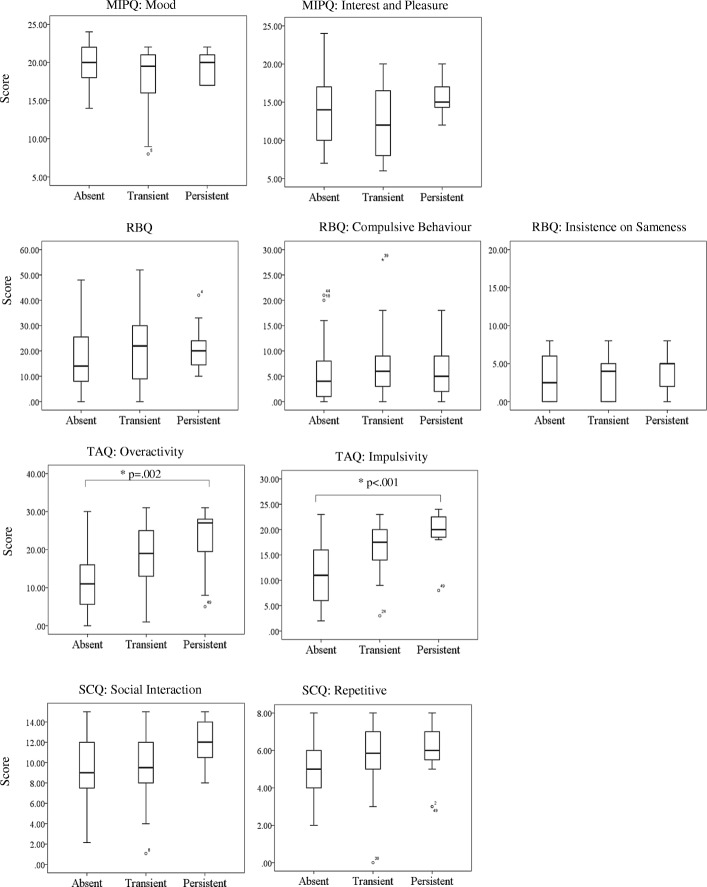
*T*_1_ MIPQ, RBQ, TAQ, and SCQ total and subscale scores for absent, transient and persistent groups

This analysis was repeated in order to assess hypothesis 4 and evaluate putative risk markers between those with self-injury and those without. Participants were categorised into three groups: absent (self-injury absent at both *T*_1_ and *T*_3_; *N* = 37 mean [SD] age in years = 13 [10], % male = 81), transient (self-injury absent at either *T*_1_ or *T*_3_; *N* = 18 mean [SD] age in years = 11 [6], % male = 83) and persistent (self-injury present at both *T*_1_ and *T*_3_; *N* = 11 mean [SD] age in years = 10 [6], % male = 73)^3^. *T*_1_ behavioural characteristics were assessed across the three groups (see Fig. 1 for the median, maximum and minimum scores and significant differences between groups). Kruskal-Wallis analyses identified significant differences between groups on measures of overactivity (*χ*^2^ (2) = 16.067, *p* < .001) and impulsivity (*χ*^2^ (2) = 20.418, *p* < .001). Pairwise post hoc analysis corrected for multiple comparisons revealed significant differences between scores in the absent and persistent self-injury groups, with the persistent group scoring significantly higher on measures of overactivity (*U* = 76, *p* = .002, *r* = 0.5) and impulsivity (*U* = 45.5, *p* < .001, *r* = 0.6).

**Fig. 2:**
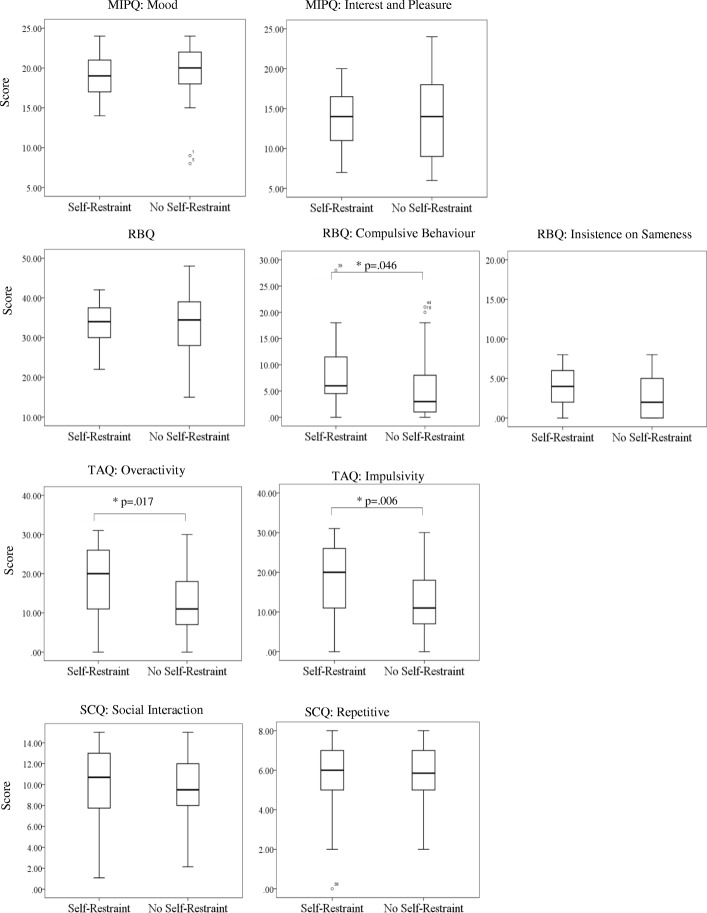
*T*_1_ MIPQ, RBQ, TAQ, and SCQ total and subscale scores for individuals with and without self-restraint behaviours at *T*_3_

In order to evaluate putative risk markers associated with the presence of self-restraint behaviours at *T*_3_, measures of *T*_1_ behavioural characteristics were assessed (see Fig. 2 for the median, maximum and minimum scores and significant differences between groups). Mann-Whitney *U* analyses identified significant differences between groups on measures of compulsive behaviours (*U* = 368, *Z* = − 1.993, *p* = .046, *r* = 0.2) overactivity (*U* = 363, *Z* = − 2.387, *p* = .017, *r* = 0.3) and impulsivity (*U* = 333, *Z* = − 2.762, *p* = .006, *r* = 0.3), with those showing self-restraint behaviours at *T*_3_ scoring higher on the *T*_1_ measures. No other significant differences were found on other any other measures.

In summary, analysis suggests a profile of behavioural characteristics that are associated with the presence of self-injury. Impulsivity and overactivity scores obtained at *T*_1_ significantly predict the presence of self-injury and self-restraint behaviours 10 years later, at *T*_3_, supporting hypothesis 4.

### Cross-sectional and longitudinal analysis summary

In order to compare both cross-sectional and longitudinal risk markers for self-injury over the 10-year data set, summary analyses are presented in Table 8. Table 8 presents effect sizes (R interpreted with Cohens *D*) of significant differences between present and absent SIB groups (cross-sectionally) and persistent and absent SIB groups (longitudinally). Data are drawn from the previously published studies [8, 24] and the analyses conducted in the present study. Analysis was also conducted for the demographic variables presented at each of the three time points; however, as none of these significantly predicted differences longitudinally they were not included in the final table. The results in Table 8 demonstrate that impulsivity and overactivity are the only behavioural variables that predict self-injury both cross-sectionally and longitudinally.

### Predictive model of risk markers for longitudinally predicting the presence of self-injury and self-restraint behaviours

**Fig. 3:**
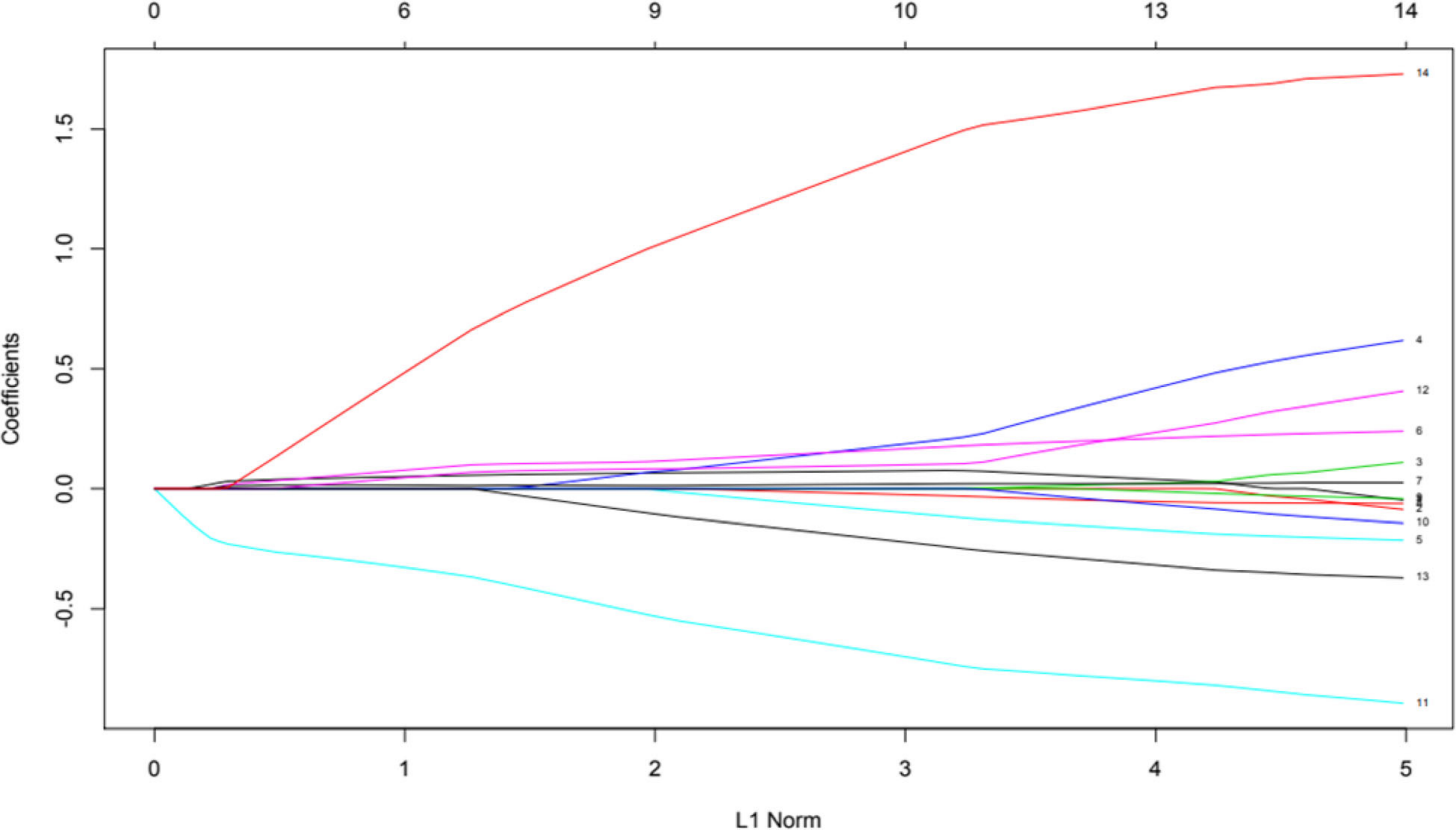
Solution path plotting Self-Injury variable coefficients against the L1 norm

**Fig. 4:**
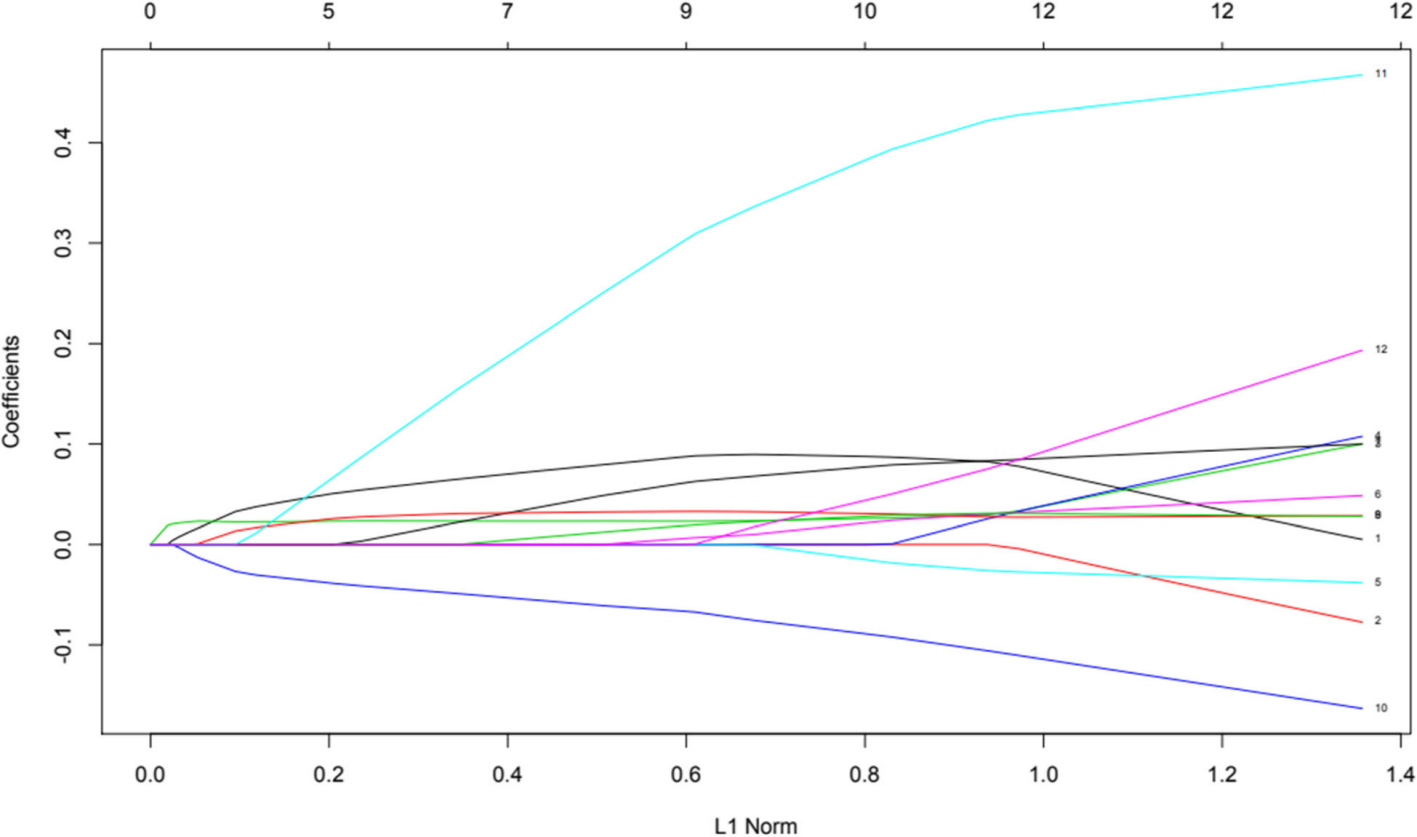
Solution path plotting self-restraint variable coefficients against the L1 norm

**Fig. 5:**
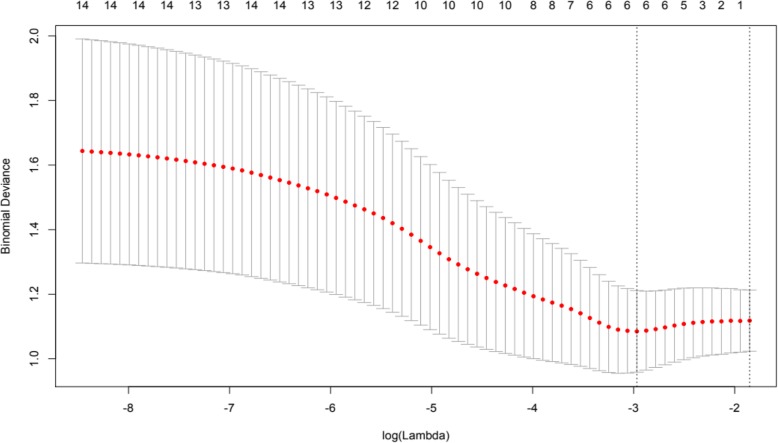
Cross-validation plot for Self-Injury predictors, estimating optimal Lambda minimum and maximum estimates using the deviance metric

**Fig. 6:**
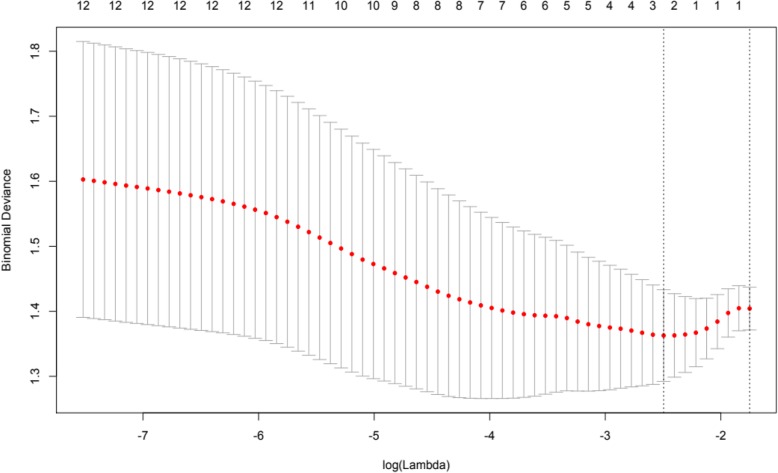
Cross-validation plot for self-restraint predictors, estimating optimal Lambda minimum and maximum estimates using the deviance metric

Finally, in order to further assess hypothesis 4 and evaluate the utility of scores obtained at *T*_1_ to predict self-injury severity and self-restraint at *T*_3_, least absolute shrinkage and selection operator (LASSO) analysis was employed. Behavioural variables collected at *T*_1_ were entered into the LASSO analysis, to control for potential multicollinearity. Outcome variables were set as *T*_3_ self-injury and *T*_3_ self-restraint in turn. As *T*_3_ self-injury severity scores were not normally distributed, responses were converted into factor variables (two levels: self-injury, no self-injury). Figures 3 and 4 present variables responding to the weight of the penalty increasing for each model. Cross-validation utilising the binomial deviance as a function of log lambda was then applied (Figs. 5 and 6). Shrinkage penalty parameters for Lambda (*λ*) were determined through tenfold cross validation [49]. All variables with zero coefficients were removed from each of the final models.

Impulsivity, interest and pleasure, stereotyped behaviour and ability at *T*_1_, as well as the presence of self-restraint at *T*_3_, were included in the final model predicting the presence of self-injury at *T*_3_. Overactivity scores from *T*_1_ were the only remaining variable predicting self-restraint at *T*_3_. Levels of predictive error as shown within cross-validation plots suggest models should be interpreted with caution, although variables remaining in the final self-injury model are supported by prior analyses.

In summary, the analysis presents two models of prediction for the presence of self-injury and self-restraint. Remaining variables presented in the model predicting self-injury support hypotheses 4 (a). The model of prediction for self-restraint fails to support hypothesis 4 (b), as variables hypothesised were converted into zero coefficients and not found to be predictive of self-restraint behaviours.

## Discussion

This study details a unique longitudinal investigation into the persistence of self-injury and self-restraint within a sample of individuals with autism over a 10-year period. The use of robust measures at each point of data collection strengthens the validity and reliability of findings. Stringent exclusion criteria and evaluation of demographic variability between those who participated and those who did not ensures that the current sample is representative of the wider non-clinical sample, further contributing to the internal validity of conclusions. The use of novel, conservative longitudinal data analysis approaches allows for unique predictive models to be proposed. Results present a robust argument for the presence of behavioural risk markers such as impulsivity and overactivity successfully predicting self-injury in autism over a 10-year period.

The results show that self-injury was persistent in 44% of individuals over 10 years, with rates of self-injury significantly decreasing from *T*_1_. Findings support cross-sectional and longitudinal literature presenting an age-related decline in the persistence of self-injury [18, 19]. Significant reductions in self-injury suggest a divergent trajectory in autism compared to those with ID, where higher persistence rates are reported, 84% over an 18-year period [18]. Current findings must also be viewed independently of research involving clinical populations, where self-injury may also be driven by elevated levels of co-morbid mood, anxiety and behavioural disorders [50]. Age-related decline in behavioural measures of autism symptomology, stereotyped behaviours and repetitive behaviours are reported for individuals with autism post adolescence [16, 20, 51]. Current findings may therefore represent a global age-related decrease in clinical behaviours for some individuals with autism. Whilst overall persistence of SIB decreased over time, it is also important to note that SIB was persistent for a significant minority (44%) of individuals with autism. Self-injury beyond the age of 20 is suggested to be a chronic behaviour requiring professional intervention [18]. Thus, these data provide support for arguments advocating early intervention to prevent the behaviour from occurring and subsequently persisting over time.

Results show significant differences between absent and persistent groups regarding access to paediatricians, with no other significant differences regarding access to other professionals. Findings are consistent with literature highlighting a considerable paucity of service use amongst individuals with intellectual disabilities [7, 52]. Individuals who engage in self-injury are considered to present a greater need for professional input to reduce such behaviours [53], however current findings suggest this need is not met, despite the persistent presence of clinically significant SIB for 10 years. It could be argued for those without self-injury, services offer a protective role in preventing the development of the behaviour. Participants were initially recruited through a parent support group, with those participating in the current study potentially representing a subsample more willing or able to interact with professionals, inflating service use data estimates. Nevertheless, even with the consideration of inflation of data within those who do not present with self-injury, the potential un-met needs for individuals with self-injury is concerning. The lack of reported access to professional services to address self-injury is proposed be a key factor in its subsequent persistence [7]; it is therefore imperative future research and policy providers investigate this issue further to encourage proactive and persistent interventions from professionals for those with self-injury.

Cross-sectional analysis of *T*_3_ characteristics associated with self-injury and self-restraint revealed significant differences in the behavioural profile for individuals presenting with these behaviours. Higher scores on measures of overactivity, impulsivity and repetitive behaviours were associated with both self-injury and self-restraint, consistent with data in other studies [23, 54]. These results support the hypothesis that impaired behavioural inhibition may drive SIB in those with autism [55]. Autism phenomenology scores were also significantly higher in individuals presenting with self-injury at *T*_3_, supporting research associating severity of autism symptomology with severe SIB [19]. The use of a standardised screening tool to score autism symptomology allows robust conclusions to be drawn and supports the clinical implications of conclusions. Findings enhance understanding of the behavioural profile associated with individuals presenting with self-injury, but also how this is differentiated for individuals without the behaviour.

Individuals who presented with self-restraint behaviour at *T*_3_ also showed significantly lower mood, interest and pleasure scores and significantly higher impulsivity scores. Self-restraint behaviours are described to serve the purpose of inhibiting severe SIB [29, 31]. Results present an emerging behavioural profile of individuals who show self-restraint. Individuals appear to be more impulsive and experience more frequent and severe self-injury. It is well-documented that painful health conditions are more common in individuals with autism, elevated for those presenting with self-injury [56]. It could be argued that lower mood occurs as a result of pain associated with the complex behavioural profile for individuals presenting with self-restraint [57]. The identification of self-restraint behaviours within the current study was limited to behavioural presence, with no record of duration or severity for individual topographies and how this may relate to mood. However, literature supporting the association of pain with elements of the presented behavioural profile suggests lower mood linked to pain is a plausible explanation [58].

Investigation of *T*_1_ behavioural markers associated with the presence of self-injury and self-restraint at *T*_3_ revealed that overactive and impulsive behaviours continue to predict self-injury and self-restraint longitudinally, as found at *T*_2_ analysis [24]. The identification of stable and reliable behavioural markers of SIB considerably enhances current understanding of mechanisms underpinning the persistence of self-injury and its age-related developmental trajectory. Furthermore, results highlight the potential positive clinical impact of identifying individuals at greater risk of developing severe self-injury. Utilising behavioural characteristics that have been identified to reliably longitudinally predict the presence of negative behaviours would allow clinical services to orient to preventative rather than solely reactive interventions [23]. The use of validated behavioural assessments at each of the time point in the present study significantly enhances the internal validity of conclusions made. Future research should attempt to corroborate findings through the employment of behavioural focussed intervention strategies, whereby intervention techniques are tailored to individual risk to ensure maximum value for both individuals and service providers.

Results present two explorative models for demographic and behavioural variables that longitudinally predict the presence of self-injury and self-restraint behaviours in turn. *T*_1_ behavioural measures that remain in the final model as having predictive value for the presence of behaviours provide support for arguments of individual characteristics influencing the developmental trajectory of self-injury and self-restraint [22]. These analyses show that impulsivity, interest and pleasure, stereotyped behaviour, social communication and adaptive functioning predict the persistence of SIB over 10 years. The novel use of regularisation techniques (LASSO analysis) represents an emerging shift within the behavioural sciences towards adopting methods of machine learning. Such analysis has the capability of producing more robust and accurate predictions when compared to traditional techniques that often overfit data and lead to inflations of error [59]. It must be noted predictive error in current models is potentially inflated by smaller sample sizes and incomplete data sets. Yet the ability of such models to identify individuals at risk of developing severe negative behaviours is not limited as these approaches are more robust than traditional regression techniques. There is benefit to be gained through the use of such novel techniques within the behavioural sciences field, expanding capabilities of analysis.

In summary, findings reveal self-injury was persistent for 44% of individuals that presented with the behaviour 10 years ago, with a robust and stable profile of behavioural characteristics associated with self-injury and self-restraint presented.

## Limitations

Small sample size may limit the population parameters drawn from statistical analysis in the current study. This may be amplified by the high attrition rates from initial *T*_1_ data collection and result in inflation of scores of measures such as service access. However, recent arguments suggest there is utility in smaller samples, offering the ability to investigate theoretical relationships at the individual participant level [60]. It must be taken into consideration when comparing the current sample with similar research that it is currently the largest longitudinal dataset utilising a non-clinical sample to explore SIB in individuals with autism and thus has significant value within its size. The current sample’s mean age from *T*_1_ to *T*_3_ stretches across early childhood to adulthood, offering significant value in its findings. Whilst future longitudinal investigations should attempt to potentially re-engage with individuals that declined the invitation to take part, the smaller sample within the current study has considerable clinical and scientific value.

Secondly, the choice of authors to utilise traditional significance statistic (*p* < .05) could be considered a limitation of analysis. However, as the nature of the research is largely exploratory, the use of a more modest estimate of significance alongside considerations of effect size was deemed sufficient in data interpretations. Where multiple comparisons have been made, stringent corrections have been put in place through the use of Bonferroni [61].

Another limitation considered by the research team is the bias seen within the socioeconomic descriptives of the sample remaining at the present time point. It is not uncommon within autism research for samples to be disproportionately representative of individuals that are highly educated and of higher socioeconomic status; however, it is something to be considered when interpreting findings proposed within the current paper.

Finally, the age suitability of measures used within the current study must also be considered. Although the SCQ is an appropriate screening measure for autism and for individuals with intellectual disabilities, questions were not adapted within the current investigation to represent the ageing sample. Literature suggests the potential benefits of modifying questions and subsequent cut-off scores to reflect samples [62]. Future research should therefore attempt to adapt questions to ensure accuracy of responses whilst maintaining the specificity of the measure.

## Conclusions

A robust and stable profile of behavioural characteristics associated with self-injury and self-restraint is presented, with their role as putative risk markers further reinforced. The ability of measurable behaviours such as overactivity and impulsivity to successfully predict individuals at greater risk of poorer outcomes over a 10-year period has significant implications for clinical interventions. Explorative models further emphasise the predictive power these behaviours have, identifying their role as mechanisms that underpin negative behaviours. Early intervention attempts should therefore target individuals considered to be at greater risk of developing severe negative behaviours and prevent them from entering into individual’s behavioural repertoire.

## Data Availability

The datasets generated and/or analysed during the current study are not publicly available. Due to the sensitive nature of the research and ethical concerns surrounding the publication of sensitive personal data, no participants were asked for consent to their data being shared.

## Abbreviations

CBQ: Challenging Behaviour Questionnaire
LASSO: Least absolute shrinkage and selection operator
MIPQ: Mood, Interest and Pleasure Questionnaire
RBQ: Repetitive Behaviour Questionnaire
SCQ: Social Communication Questionnaire
SIB: Self-Injurious Behaviour
TAQ: The Activity Questionnaire

## Footnotes

1 Relevant professionals were considered to be any of the following: paediatrician, clinical psychologist, clinical geneticist, GP, psychiatrist, educational psychologist or significant other.

2 Effect size *r* was employed as an alternative to standard difference statistics when assumptions are violated [47, 48]. Effect sizes presented are interpreted with Cohen’s *d*

3 Total sample 66 due to missing data of one participant
